# Intravitreal bevacizumab as an adjunct to vitrectomy in advanced Eales’ disease

**DOI:** 10.1007/s12348-011-0049-3

**Published:** 2011-11-17

**Authors:** Meenakshi Thakar, Naina R. Bamrolia, Usha Kaul Raina, Basudeb Ghosh

**Affiliations:** Maulana Azad Medical College, New Delhi, India

**Keywords:** Bevacizumab, Eales’ disease, Tractional retinal detachment

## Abstract

**Purpose:**

This study aimed to report the use of intravitreal bevacizumab as an adjunctive treatment in two cases of advanced Eales’ disease with vitreous haemorrhage and tractional retinal detachment, prior to vitreoretinal surgery.

**Method:**

In two patients presenting with vitreous haemorrhage, retinal neovascularisation and localised tractional retinal detachment, 1.25 mg of intravitreal bevacizumab was injected prior to vitrectomy, membrane peeling and endolaser photocoagulation of retina.

**Result:**

Regression of the retinal neovascularisation with resolution of dye leakage on fluoroscein angiography was observed in both cases. Membrane peeling could be performed with minimal bleeding during vitreoretinal surgery in both cases.

**Conclusion:**

Bevacizumab may be a possible adjunctive treatment to vitreoretinal surgery for the management of Eales’ disease with tractional retinal detachment.

## Introduction

Eales’ disease although occurring worldwide is encountered commonly in the Indian subcontinent. It affects healthy young adults aged 20–30 years with a male preponderance [1]. The characteristic clinical features include occlusive peripheral retinal vasculitis leading to capillary non-perfusion and ischemia followed by neovascularisation of the disc and retina and subsequently recurrent vitreous haemorrage and tractional retinal detachment. Photocoagulation is the mainstay of therapy in the proliferative stage of Eales’ disease [2].

Recently, intravitreal bevacizumab has been explored as a possible therapeutic modality in the management of early stages of Eales’ disease [2–5]. We report two cases of Eales’ disease with tractional retinal detachment where bevacizumab was used intravitreally as an adjunctive treatment prior to vitreoretinal surgery. To our knowledge, this is probably the first report where adjunctive bevacizumab used preoperatively in a relatively advanced stage of the disease facilitated the removal of fibrovascular membranes with minimal intra- and post-operative complications.

## Case 1

**Fig. 1:**
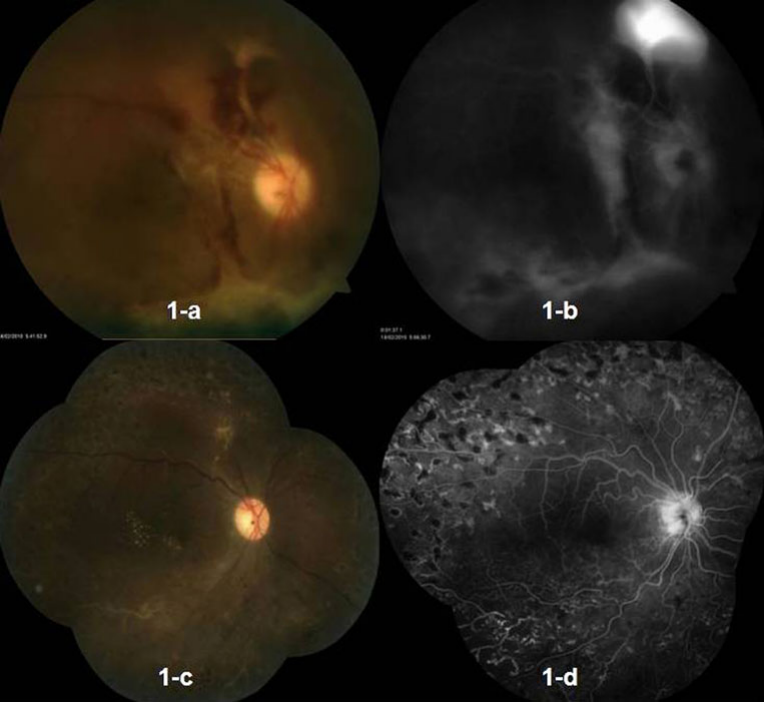
Fundus photograph and fluorescein angiogram (FA) of the right eye of Case 1 at presentation (**a** and **b**) and after vitreoretinal surgery (**c** and **d**). **a** Disc neovascularisation, retinal neovascularisation superior to the disc, presence of fibrovascular band temporal to the disc and inferior tractional retinal detachment with inferior vitreous haemmorhage. **b** (FA): Leakage of dye from the retinal neovascularisation superior to the disc and staining of the fibrovascular band temporal to the disc. **c** Well-settled retina with endolaser marks. **d** (FA): Absence of dye leakage with complete regression of neovacularisation and adequately lasered peripheral retina

A 28-year-old female presented with complaint of sudden, painless, progressive diminution of vision in her right eye since last 10 months. On examination of the right eye, the best corrected visual acuity (BCVA) was counting finger at 2 m. Anterior segment was normal on slit lamp biomicroscopy. The intraocular pressure was also normal. Fundus evaluation revealed retinal and disc neovascularisation with the presence of a fibrovascular band temporal to the disc. There was vitreous haemorrage and tractional retinal detachment inferiorly in the retina. Vitreous haemorrhage and inferior tractional retinal detachment were confirmed further on ultrasound B scan. Leakage of dye from the retinal neovascularisation superior to the disc and staining of the fibrovascular band temporal to the disc was evident on fluorescein angiogram (FA) (Fig. 1a, b). Peripheral capillary non-perfusion areas were noted in the superior periphery of retina. The left eye was phthisical following trauma 10 years back. Systemic examination, past medical and drug history were unremarkable. Investigations to rule out secondary vasculitis revealed normal haemogram, erythrocyte sedimentation rate, coagulation profile and blood sugar (fasting and post-prandial). Rheumatoid factor (RF) and Venereal Disease Research Laboratory (VDRL) test were negative. Antinuclear antibody (ANA) was not detected. Test for HIV I and HIV II was negative. Sickle cell preparation was normal. Mantoux test was negative. The chest radiograph was normal. A diagnosis of Eales’ disease was made. An informed consent was obtained from the patient for bevacizumab injection after the “off-label” status of bevacizumab and its possible systemic and ocular complications were discussed in detail. From a commercially available 4 ml vial containing 100 mg bevacizumab (Avastin, Genetech Inc, CA, USA), 0.05 ml (containing 1.25 mg) was withdrawn into in a tuberculin syringe using aseptic techniques. After preparing the eye in a standard fashion using 5% povidone/iodine, an eyelid speculum was used to stabilise the eyelids, and bevacizumab (0.05 ml) was injected 4 mm posterior to the limbus, through the pars plana with a 30-gauge needle under subconjunctival lignocaine. Following the injection, intraocular pressure and retinal artery perfusion were confirmed, and patient was instructed to administer topical ofloxacin eyedrops for 7 days.

Six weeks post-injection, vitreoretinal surgery was performed with membrane peeling and endolaser photocoagulation. Membrane peeling was easily performed without the need of diathermy during surgery. On the first post-operative day, adequate fundal glow indicated proper haemostasis without post-operative oozing. At 2 weeks follow-up, BCVA in the right eye improved to 6/36 (Snellen chart). On fundus evaluation, there was absence of retinal or disc neovascularisation. Fluorescein angiogram (Fig. 1c, d) revealed absence of leakage from regressed vessels and an appropriately lasered peripheral retina. Staining of the inferotemporal vessel seen in the late phase indicated healed vasculitis. At 6 months follow-up, BCVA was maintained without recurrence of neovascularisation or vitreous haemorrhage.

## Case 2

**Fig. 2:**
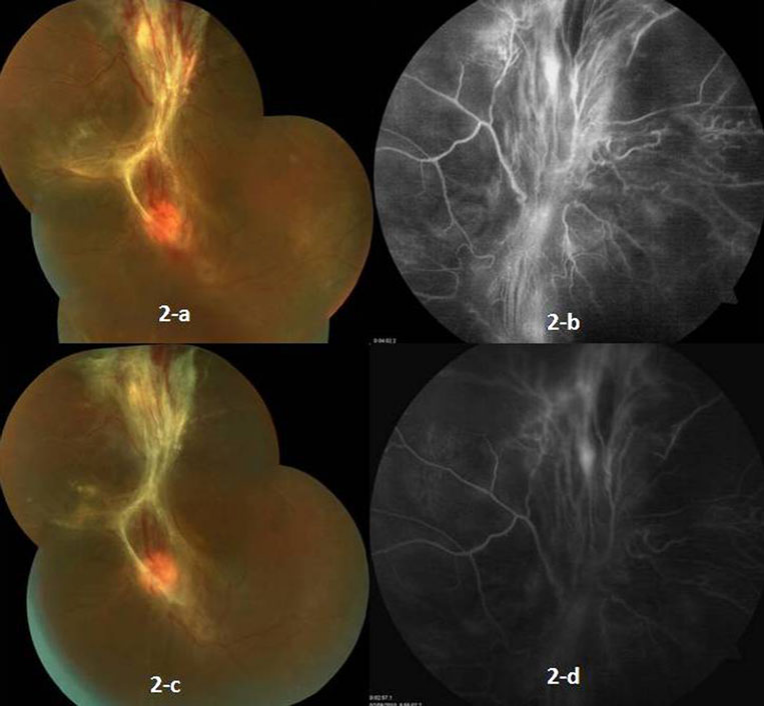
Fundus photograph and fluorescein angiogram of the left eye of Case 2 at presentation (**a** and **b**) and 20 days after bevacizumab injection (**c** and **d**). **a** Presence of fibrovascular frond extending from the disc superiorly and superior tractional retinal detachment. **b** (FA): Leakage of dye from neovascularisation superior to the disc and staining of fibrovascular band. **c** Marked regression of the new vessels. **d** (FA): Staining of the glial tissue without any dye leakage

A 19-year-old male presented with complaints of sudden, painless, progressive diminution of vision in the left eye for last 4 months. The BCVA in the left eye was counting finger at 2 m. Anterior segment was normal on slit lamp biomicroscopy. Fundus examination showed fibrovascular frond extending from the disc superiorly along with superior tractional retinal detachment and inferior vitreous haemorrage. Leakage of dye from the neovascular frond was revealed in FA (Fig. 2a, b). The right eye was normal with visual acuity of 6/6. Systemic examination, past medical and drug history were unremarkable. Haematological investigations and other tests conducted to rule out secondary vasculitis including haemogram, erythrocyte sedimentation rate, coagulation profile, blood sugar and sickle cell preparation were normal. RF, ANA, VDRL, HIV I, HIV II and Mantoux test were negative. The chest radiograph was also normal. A diagnosis of Eales’ disease was made. An informed written consent was obtained and bevacizumab was injected intravitreally (1.25 mg/0.05 ml) through pars plana in a similar fashion as the previous case with a 30-gauge needle under all aseptic precautions.

Four days post-injection, fundus evaluation and fluorescein angiogram revealed mild regression of new vessels and persistence of the dye leakage. Twenty days later, repeat FA showed staining of fibroglial tissue without any leakage of dye from the regressed vessels (Fig. 2c, d). On 21st day post-injection, a three-port pars plana vitrectomy was performed along with membrane peeling followed by endolaser photocoagulation and silicone oil tamponade. There was minimal bleeding during membrane peeling. Post-operatively, the patient was prescribed with ofloxacin drops four times a day and prone positioning was advised. On the first post-operative day, there was adequate fundal glow and the retina appeared settled with silicone oil in situ and endolaser marks could be visualised. Four weeks later, BCVA improved to 6/60.

## Discussion

It is well established that retinal ischaemia stimulates the production of vascular endothelial growth factor (VEGF), a key mediator of angiogenesis. Massive expression of VEGF in the neovascular membranes in Eales’ disease has also been reported previously [6, 7]. Bevacizumab, a humanised monoclonal antibody against VEGF, is currently emerging as an effective off-label treatment for a variety of ocular disorders such as neovascular age-related macular degeneration, macular edema secondary to central retinal vein occlusion and proliferative diabetic retinopathy [8]. However, a literature search revealed only few anecdotal reports on the use of intravitreal bevacizumab in Eales’ disease [2–5]. In all the reports, bevacizumab had been used in early stages of the disease without retinal detachment. In contrast, our cases had fairly advanced Eales’ disease with tractional retinal detachment, and bevacizumab was effective in promoting regression of neovascularisation, thus facilitating membrane dissection by reducing intra-operative bleeding. Additionally, due to better visibility during surgery, the chances of causing iatrogenic retinal breaks were also reduced. Although photocoagulation is the preferred modality of treatment in the proliferative stage of Eales’ disease, it could not be performed in both our cases due to the presence of dispersed vitreous haemorrhage and tractional retinal detachment. Nevertheless, there was no recurrence of neovascularisation at 6 months follow-up in our first patient, and at the time of writing this report, the second patient had been followed up for 2 months without recurrence of neovascularisation.

In both our cases, sequential treatment with bevacizumab followed by vitrectomy was beneficial and associated with fair visual outcome, despite the presence of tractional retinal detachment. However, the appropriate time interval between bevacizumab injection and vitreoretinal surgery in the presence of tractional retinal detachment is not established. The minimum concentration (500 ng/ml) of intravitreal bevacizumab (1.25 mg) that completely inhibits VEGF-A-induced endothelial cell proliferation is reportedly maintained for 6–7 weeks, after which repeat administration of bevacizumab may be required [9]. We performed vitreoretinal surgery within 3–6 weeks after injection of bevacizumab as the anti-angiogenic effect of drug was expected to be maintained during this period while allowing reasonably sufficient time for regression of neovascularisation. Additionally, in our first case, non-recurrence of neovascularisation at 6 months follow-up suggests that intermediate-term control is achievable with intravitreal bevacizumab and vitreoretinal surgery, even with a background of ongoing ischemic disease. Although recent studies have strengthened the favourable short-term safety profile of intravitreal bevacizumab, long-term safety of intravitreal bevacizumab is still unknown [10]. Therefore, prudent clinical judgement and caution should be exercised with the off-label use of intravitreal bevacizumab, as there have been few reports of progression of tractional retinal detachment in patients with severe proliferative diabetic retinopathy [11].

To summarise, the use of intravitreal bevacizumab provided a relatively clean surgical field and facilitated membrane dissection with reduced chance of iatrogenic retinal break. Therefore, bevacizumab may have potential as an adjunctive treatment to vitreoretinal surgery for the management of Eales’ disease with tractional retinal detachment. However, well-designed randomised controlled trials are essential before such a use can be unequivocally established.
